# Prognostic Value of TSPO PET Before Radiotherapy in Newly Diagnosed IDH–Wild-Type Glioblastoma

**DOI:** 10.2967/jnumed.122.265247

**Published:** 2023-10

**Authors:** Nathalie L. Albert, Debie V. Nelwan, Daniel F. Fleischmann, Stefanie Quach, Katharina von Rohr, Lena Kaiser, Nico Teske, Lena M. Unterrainer, Laura M. Bartos, Viktoria C. Ruf, Matthias Brendel, Markus J. Riemenschneider, Christian Wetzel, Jochen Herms, Rainer Rupprecht, Niklas Thon, Joerg-Christian Tonn, Claus Belka, Peter Bartenstein, Louisa von Baumgarten, Maximilian Niyazi, Marcus Unterrainer, Adrien Holzgreve

**Affiliations:** 1 Department of Nuclear Medicine, LMU University Hospital, LMU Munich, Munich, Germany;; 2 German Cancer Consortium, Partner Site Munich, German Cancer Research Center, Munich, Germany;; 3 Bavarian Cancer Research Center, Erlangen, Germany;; 4 Department of Radiation Oncology, LMU University Hospital, LMU Munich, Munich, Germany;; 5 Department of Neurosurgery, LMU University Hospital, LMU Munich, Munich, Germany;; 6 Institute of Neuropathology, Faculty of Medicine, LMU Munich, Munich, Germany;; 7 SyNergy, University of Munich, Munich, Germany;; 8 German Center for Neurodegenerative Diseases, Munich, Germany;; 9 Department of Neuropathology, Regensburg University Hospital, Regensburg, Germany;; 10 Department of Psychiatry and Psychotherapy, University of Regensburg, Regensburg, Germany; and; 11 Department of Radiology, LMU University Hospital, LMU Munich, Munich, Germany

**Keywords:** prognostication, survival, glioma

## Abstract

The 18-kDa translocator protein (TSPO) is gaining recognition as a relevant target in glioblastoma imaging. However, data on the potential prognostic value of TSPO PET imaging in glioblastoma are lacking. Therefore, we investigated the association of TSPO PET imaging results with survival outcome in a homogeneous cohort of glioblastoma patients. **Methods:** Patients were included who had newly diagnosed, histologically confirmed isocitrate dehydrogenase (IDH)–wild-type glioblastoma with available TSPO PET before either normofractionated radiotherapy combined with temozolomide or hypofractionated radiotherapy. SUV_max_ on TSPO PET, TSPO binding affinity status, tumor volumes on MRI, and further clinical data, such as *O*^6^-alkylguanine DNA methyltransferase (*MGMT*) and telomerase reverse transcriptase (*TERT*) gene promoter mutation status, were correlated with patient survival. **Results:** Forty-five patients (median age, 63.3 y) were included. Median SUV_max_ was 2.2 (range, 1.0–4.7). A TSPO PET signal was associated with survival: High uptake intensity (SUV_max_ > 2.2) was related to significantly shorter overall survival (OS; 8.3 vs. 17.8 mo, *P* = 0.037). Besides SUV_max_, prognostic factors for OS were age (*P* = 0.046), *MGMT* promoter methylation status (*P* = 0.032), and T2-weighted MRI volume (*P* = 0.031). In the multivariate survival analysis, SUV_max_ in TSPO PET remained an independent prognostic factor for OS (*P* = 0.023), with a hazard ratio of 2.212 (95% CI, 1.115–4.386) for death in cases with a high TSPO PET signal (SUV_max_ > 2.2). **Conclusion:** A high TSPO PET signal before radiotherapy is associated with significantly shorter survival in patients with newly diagnosed IDH–wild-type glioblastoma. TSPO PET seems to add prognostic insights beyond established clinical parameters and might serve as an informative tool as clinicians make survival predictions for patients with glioblastoma.

## Visual Abstract

**Figure:**
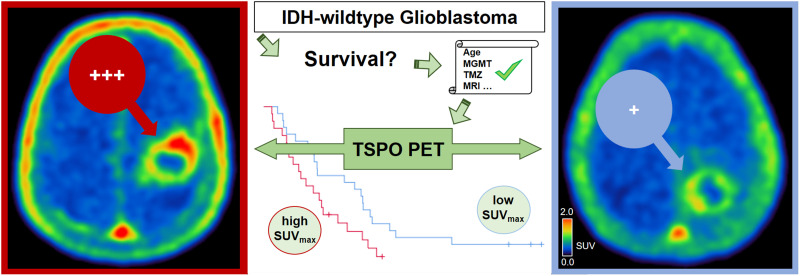

Glioblastoma is the most frequent malignant primary brain tumor in adults, and diagnosis is associated with a short life expectancy (1). Although median overall survival (OS) can reach up to 4 y with a molecular profile favorable to adapted chemotherapy regimens, most glioblastoma patients have a shorter survival period (1). Overall, successful treatment options remain limited and there is a need to explore new targets for both diagnostics and therapy of glioblastoma.

The 18-kDa translocator protein (TSPO) is a ubiquitous mitochondrial protein that is gaining recognition as a relevant target in glioblastoma (2). TSPO has been widely studied in neuroinflammatory diseases because it is considered a marker for activated microglia. However, evidence is growing that TSPO also intervenes in multiple pathophysiologic processes in glioblastoma, including proliferation, invasiveness, and resistance to apoptosis (2). TSPO ligands can be radiolabeled and therefore are suitable for in vivo imaging by PET. As a result, TSPO PET has been used in several neurologic disease areas with an immune-mediated component well beyond primary neuroinflammatory disorders, such as neurodegeneration (3). The coincidence of genuine tumor cell–associated TSPO expression and neuroinflammation in glioblastoma underscores that TSPO PET could be a valuable imaging modality in glioblastoma patients as well.

Preliminary in vivo studies or case reports using TSPO imaging in glioma were promising, revealing high tumoral tracer uptake, especially in glioblastoma (4–7). However, studies investigating a clinical benefit of TSPO PET in neurooncology remain scarce, and data on its potential prognostic value are lacking. Therefore, we investigated the association of TSPO PET imaging results with survival outcome in a homogeneous cohort of histologically proven isocitrate dehydrogenase (IDH)–wild-type glioblastoma before radiotherapy.

## MATERIALS AND METHODS

### Patients

Patients were included who had newly diagnosed, neuropathologically confirmed glioblastoma and available TSPO PET before normofractionated (2-Gy dose per fraction and 60-Gy total dose) or hypofractionated (2.67-Gy dose per fraction and 40.05-Gy total dose) radiotherapy. In all cases of tumor resection, only postoperative PET images were used. Normofractionated radiotherapy was combined with temozolomide in all cases. Patients undergoing hypofractionated radiotherapy received concomitant temozolomide if recommended by the interdisciplinary tumor board, primarily based on the molecular tumor profile. The local ethics committee gave permission to perform the study (Institutional Review Board 601-16 and 17-457). All patients signed an informed consent form.

### Histopathologic and Molecular Diagnostics

Patients received either stereotactic biopsy or microsurgical tumor resection according to clinical routine. Histologic and molecular genetic assessments were performed according to clinical routine, and all cases were classified on the 2021 World Health Organization’s Classification of Tumours of the Central Nervous System (8).

### Polymorphism Genotyping

The evaluation of patients’ TSPO binding affinity status—low-affinity binding (LAB), medium-affinity binding (MAB), or high-affinity binding (HAB)—was conducted as previously described (4).

### TSPO PET Acquisition and Assessment

TSPO PET was performed on a Biograph-64 PET/CT scanner (Siemens Healthineers). The TSPO radioligand [^18^F]GE-180 was synthesized as previously described (4). [^18^F]GE-180 was intravenously injected (181 ± 17 MBq). Low-dose CT was performed for attenuation correction. PET emission data were recorded 60–80 min after injection, and the summation images taken 60–80 min after injection were used for image analyses. Reconstruction parameters were applied as previously described (4). For evaluation of PET images, tumoral SUV_max_ was assessed.

### MRI

As part of the clinical routine, all patients received MRI scans for radiation treatment planning (median time between PET and MRI, 12 d). In all cases of tumor resection, only postoperative images were used. Axial T1-weighted sequences before and after intravenous injection of 0.1 mmol/kg gadobenate dimeglumine contrast agent (MultiHance; BraccoImaging) were analyzed to measure the total contrast-enhancing tumor, and T2 or fluid-attenuated inversion recovery (FLAIR) sequences were used to measure non–contrast-enhancing tumors. Tumor volumes were manually delineated as defined by the Advisory Committee for Radiation Oncology Practice of the European Society for Radiotherapy and Oncology guidelines using the institutional imaging software (BrainLab Smartbrush; BrainLab) (9). In cases of multifocal disease, each focus was quantified separately and summed together. In patients undergoing microsurgical tumor resection before radiotherapy, we ensured that postoperative T2 or FLAIR abnormalities were not surgically induced edema or ischemia by reviewing diffusion-weighted imaging sequences.

### Clinical Evaluation, Tumor Progression, and Clinical Endpoints

Regular clinical follow-up consisted of clinical evaluation and MRI evaluation every 3 mo, supplemented by *O*-(2-[^18^F]-fluoroethyl)-l-tyrosine ([^18^F]FET) PET if appropriate. Tumor progression was defined according to Response Assessment in Neuro-Oncology criteria (10): at least 25% diameter increase of the contrast-enhancing lesion; a significant increase in the T2 or FLAIR nonenhancing lesion; new lesions; clinical deterioration, probably caused by the tumor and no other causes apart from it; failure to show up; or death.

The clinical primary endpoint of this study was OS, defined as the time from first diagnosis until death of the patient. The secondary endpoint was progression-free survival (PFS), defined as the time from first diagnosis until tumor progression.

### Statistics

SPSS version 26 (IBM) was used for the statistical analysis. The Shapiro–Wilk test was used to assess normal distribution. The Wilcoxon signed-rank test, the Mann–Whitney *U* test or Kruskal–Wallis H test, and finally the Dunn–Bonferroni post hoc test were used to investigate group differences. Linear bivariate association between variables was obtained using the Pearson correlation. For survival analysis, continuous parameters underwent median split dichotomization. Univariate survival analysis consisted of the Kaplan–Meier estimator and log-rank test. Parameters found to be prognostic in univariate analysis were subsequently included in multivariate survival analysis using the Cox proportional hazards model. Statistical significance was defined as a 2-tailed *P* value of less than 0.05.

## RESULTS

### Patient Characteristics

Forty-five patients were included. Patient characteristics are displayed in Table 1. Cycles of concomitant and adjuvant temozolomide are given in Table 2.

**TABLE 1.** Patient Characteristics

Characteristic	Data
Age (y)	63.3 (30.6–84.2)
Sex	
Male	27 (60.0%)
Female	18 (40.0%)
*MGMT* promoter methylation status	
Methylated	12 (26.7%)
Unmethylated	33 (73.3%)
*TERT* promoter mutation status	
Mutant	39 (86.7%)
C250T mutation	15 (53.3%)
C228T mutation	24 (33.3%)
Wild-type	6 (13.3%)
KPS	80% (60–100%)
Mode of radiotherapy	
Conventional	23 (51.1%)
Hypofractionated	22 (48.9%)
Mode of surgery	
Stereotactic biopsy	35 (77.8%)
Microsurgical resection	10 (22.2%)
Contrast enhancement	
Yes	40 (88.9%)
No	5 (11.1%)
CE-T1w MRI volume (mL)	11.1 (0.0–112.6)
T2w MRI volume (mL)	40.9 (0.0–272.0)
TSPO polymorphism genotype	
LAB	6 (13.3%)
MAB	15 (33.3%)
HAB	18 (40.0%)
Not specified	6 (13.3%)
SUV_max_	2.2 (1.0–4.7)

*MGMT* = *O*^6^-alkylguanine DNA methyltransferase gene; *TERT* = telomerase reverse transcriptase gene; KPS = Karnofsky performance status scale; CE-T1w = contrast-enhanced T1-weighted; T2w = T2-weighted.

Qualitative data are number and percentage; continuous data are median and range.

**TABLE 2.** Univariate Survival Analyses

	OS		PFS	
Parameter	Median OS	Significance*	Median PFS	Significance*
Age		***P* = 0.046**		***P* = 0.014**
Median < 63 y, *n* = 22	16.33 (9.39–23.27)		10.41 (7.07–13.76)	
Median ≥ 63 y, *n* = 23	9.69 (7.28–12.11)		5.88 (3.46–8.30)	
Sex		*P* = 0.330		*P* = 0.259
Male, *n* = 27	10.48 (2.90–18.06)		6.41 (3.12–9.70)	
Female, *n* = 18	10.84 (8.32–13.37)		9.69 (5.94–13.45)	
*MGMT* promoter methylation status		***P* = 0.032**		***P* = 0.028**
Methylated, *n* = 12	19.38 (3.16–35.61)		10.84 (4.60–17.09)	
Unmethylated, *n* = 33	10.42 (8.60–12.23)		7.26 (4.49–10.03)	
*TERT* promoter mutation status		*P* = 0.939		*P* = 0.896
Mutant, *n* = 39	10.42 (8.04–12.79)		7.69 (4.99–10.38)	
Wild-type, *n* = 6	11.30 (6.93–15.70)		8.35 (3.02–13.67)	
KPS		*P* = 0.117		*P* = 0.104
Median < 80, *n* = 26	8.61 (4.83–12.38)		6.21 (4.32–8.10)	
Median ≥ 80, *n* = 19	17.77 (13.15–22.40)		9.69 (7.54–11.84)	
Mode of radiotherapy		*P* = 0.105		*P* = 0.175
Conventional, *n* = 23	16.33 (11.48–21.18)		8.90 (7.41–10.40)	
Hypofractionated, *n* = 22	6.83 (0.72–12.95)		5.29 (2.61–7.97)	
Conventional radiochemotherapy		*P* = 0.081		***P* = 0.024**
≥ 1 cycle adjuvant temozolomide, *n* = 12	19.29 (13.99–24.58)		10.42 (8.93–11.90)	
No adjuvant temozolomide, *n* = 11	9.86 (3.67–16.04)		6.21 (5.50–6.92)	
Hypofractionated radiotherapy		*P* = 0.064		*P* = 0.100
With concomitant temozolomide, *n* = 13	10.84 (0.0–26.94)		8.08 (1.21–14.96)	
Without concomitant temozolomide, *n* = 9	6.83 (1.16–12.51)		5.29 (3.98–6.60)	
Mode of surgery		*P* = 0.118		*P* = 0.443
Stereotactic biopsy, *n* = 35	9.86 (7.31–12.41)		6.83 (3.90–9.77)	
Microsurgical resection, *n* = 10	19.29 (16.77–21.80)		8.35 (2.61–7.97)	
Contrast enhancement		*P* = 0.070		*P* = 0.419
Yes, *n* = 40	9.86 (8.63–11.08)		6.83 (4.54–9.13)	
No, *n* = 5	21.22 (17.42–25.03)		10.61 (7.79–13.43)	
CE-T1w MRI volume		*P* = 0.289		*P* = 0.372
Median < 11.1 mL, *n* = 23	15.77 (7.90–23.64)		8.90 (7.52–10.29)	
Median ≥ 11.1 mL, *n* = 22	6.21 (0.70–11.72)		5.65 (3.65–7.65)	
T2w MRI volume		***P* = 0.031**		*P* = 0.118
Median < 40.9 mL, *n* = 23	17.77 (6.20–29.34)		8.90 (6.47–11.33)	
Median ≥ 40.9 mL, *n* = 22	6.83 (1.32–12.35)		5.65 (2.90–8.41)	
TSPO polymorphism genotype		*P* = 0.360		*P* = 0.333
LAB	10.84 (8.91–12.77)		9.69 (5.91–13.48)	
MAB	17.77 (7.99–27.56)		8.90 (4.76–13.05)	
HAB	8.28 (0.78–15.79)		5.29 (3.72–6.86)	
SUV_max_		***P* = 0.037**		*P* = 0.333
Median < 2.2, *n* = 23	17.77 (4.40–31.14)		8.41 (7.56–9.23)	
Median ≥ 2.2, *n* = 22	8.28 (3.67–12.89)		6.83 (3.63–10.04)	

* Bold font highlights the statistically significant associations.

*MGMT* = *O*_6_-alkylguanine DNA methyltransferase gene; *TERT* = telomerase reverse transcriptase gene; KPS = Karnofsky performance status scale; CE-T1w = contrast-enhanced T1-weighted; T2w = T2-weighted.

Data in parentheses are 95% CI.

Median PFS and OS for the overall group were 8.1 mo (95% CI, 6.0–10.2 mo) and 10.8 mo (95% CI, 8.5–13.2 mo), respectively. At the last follow-up, 43 of 45 patients (95.6%) had experienced tumor progression and 40 of 45 patients (88.9%) had died. Of the remaining patients, 1 of 5 was lost to follow-up and 4 of 5 were alive at the last follow-up. Progression was attributable to the following criteria defined by the Response Assessment in Neuro-Oncology Working Group: increase of a contrast-enhancing lesion or a T2 or FLAIR nonenhancing lesion in 14 of 43 patients (32.6%), new contrast enhancement outside the radiation field in 5 of 43 patients (11.6%), failure to return for evaluation because of death or deteriorating condition in 24 of 43 patients (55.8%), and no patients lost to follow-up regarding tumor progression.

### TSPO PET Findings in Correlation to TSPO Binding Affinity Status, MRI Findings, and Molecular and Clinical Parameters

Median SUV_max_ for the overall group was 2.2 (range, 1.0–4.7). TSPO polymorphism genotyping results are given in Table 1. Median SUV_max_ was highest in LAB, which was 2.6 (range, 2.4–3.3), followed by MAB of 2.2 (range, 1.0–3.8) and HAB of 2.0 (range, 1.5–4.7; *P* = 0.026).

Contrast enhancement in MRI was absent for 5 patients, all of whom showed a low tumoral TSPO PET signal with SUV_max_ of less than 2.2; the median uptake in those patients was SUV_max_ of 1.8 (range, 1.5–2.1). Otherwise, there were no significant differences between the groups with SUV_max_ greater or less than the median value in PET (all *P* > 0.05; Table 3). SUV_max_ did not correlate with contrast-enhanced T1-weighted MRI volume (*r* = 0.092, *P* = 0.547) or T2-weighted MRI volume (*r* = 0.025, *P* = 0.872).

**TABLE 3.** Comparison of Patients with Low Versus High Tumoral Uptake on TSPO PET

Characteristic	SUV_max_ < 2.2, *n* = 23	SUV_max_ ≥ 2.2, *n* = 22	Significance*
Age (y)	62.6 (51.2–80.4)	63.9 (30.6–84.2)	*P* = 0.910
Sex			*P* = 0.270
Male	12 (52.2%)	15 (68.2%)	
Female	11 (47.8%)	7 (31.8%)	
*MGMT* promoter methylation status			*P* = 0.563
Methylated	7 (30.4%)	5 (22.7%)	
Unmethylated	16 (69.6%)	17 (77.3%)	
*TERT* promoter mutation status			*P* = 0.954
Mutant	20 (86.9%)	19 (86.4%)	
C250T mutation	9 (39.1%)	6 (27.3%)	
C228T mutation	11 (47.8%)	13 (59.1%)	
Wild-type	3 (13.0%)	3 (13.6%)	
KPS	80% (60–100%)	80% (60–90%)	*P* = 0.161
Mode of surgery			*P* = 0.528
Stereotactic biopsy	17 (73.9%)	18 (81.8%)	
Microsurgical resection	6 (26.1%)	4 (18.2%)	
Mode of radiotherapy			*P* = 0.185
Conventional	14 (60.9%)	9 (40.9%)	
Hypofractionated	9 (39.1%)	13 (59.1%)	
Concomitant temozolomide			*P* = 0.266
Yes	19 (82.6%)	15 (68.2%)	
No	4 (17.4%)	7 (31.8%)	
Contrast enhancement			***P* = 0.022**
Yes	18 (78.3%)	22 (100.0%)	
No	5 (21.7%)	0 (0.0%)	
CE-T1w MRI volume (mL)	10.2 (0.0–112.6)	14.5 (0.4–78.6)	*P* = 0.188
T2w MRI volume (mL)	35.2 (0.0–272.0)	56.2 (0.4–168.5)	*P* = 0.188

* Bold font highlights the statistically significant associations.

*MGMT* = *O*_6_-alkylguanine DNA methyltransferase gene; *TERT* = telomerase reverse transcriptase gene; KPS = Karnofsky performance status scale; CE-T1w = contrast-enhanced T1-weighted; T2w = T2-weighted.

Qualitative data are number and percentage; continuous data are median and range.

### Association of TSPO PET Findings and Clinical Parameters with Survival

**FIGURE 1.**
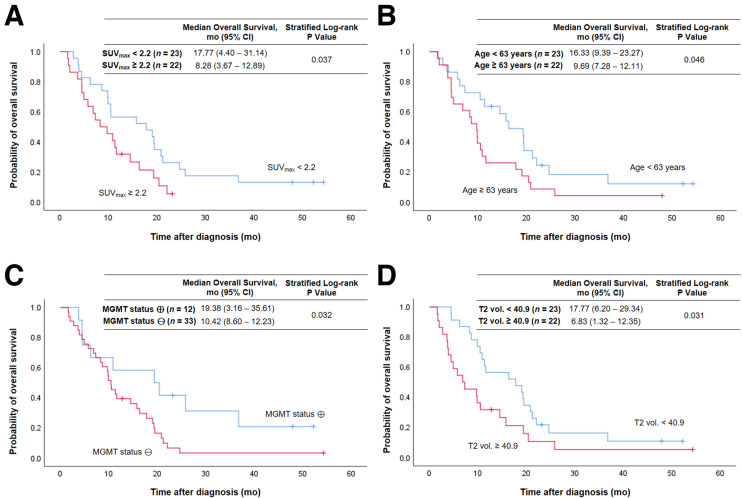
Kaplan–Meier curves of OS for entire patient group using median split of SUV_max_ (A), age (B), *MGMT* promoter methylation status (C), and median split of tumor volume on T2-weighted MRI (T2 vol.) (D). ⊕ = methylated; ⊖ = unmethylated.

**FIGURE 2.**
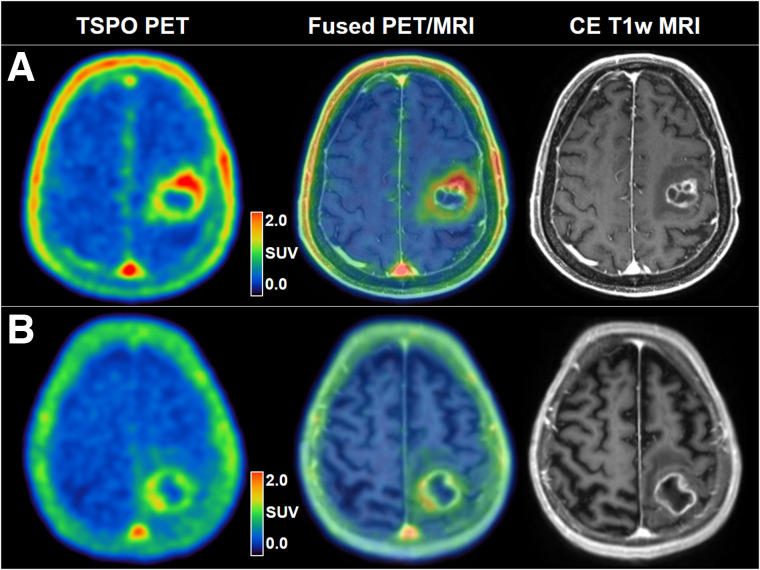
(A) 76-y-old male patient with left precentral IDH–wild-type glioblastoma (*MGMT* promoter-methylated, telomerase reverse transcriptase promoter C228T mutation; TSPO HAB) before hypofractionated radiotherapy and temozolomide chemotherapy. Tumoral TSPO radioligand uptake was high (SUV_max_, 2.4), and survival was short (OS, 4.5 mo). (B) 71-y-old female patient with left postcentral–parietal IDH–wild-type glioblastoma (*MGMT* promoter-methylated, telomerase reverse transcriptase promoter C250T mutation; TSPO MAB) before hypofractionated radiotherapy and temozolomide chemotherapy. Tumoral TSPO radioligand uptake was low (SUV_max_, 1.8), and survival was long (OS, 25.8 mo). CE-T1w = contrast-enhanced T1-weighted.

Tumoral uptake on TSPO PET was associated with OS (Fig. 1). High SUV_max_ was related to significantly shorter OS (8.3 vs. 17.8 mo, *P* = 0.037) (Fig. 1A). Figure 2 illustrates a case with high tumoral TSPO radioligand uptake associated with short survival (Fig. 2A) and a case with low tumoral TSPO radioligand uptake associated with long survival (Fig. 2B).

Besides SUV_max_, age (*P* = 0.046) (Fig. 1B), *O*^6^-alkylguanine DNA methyltransferase (*MGMT*) promoter methylation status (*P* = 0.032) (Fig. 1C), and T2-weighted MRI volume (*P* = 0.031) (Fig. 1D) were prognostic for OS. All results of the univariate analyses for OS and PFS are shown in Table 2.

In the multivariate survival analysis, SUV_max_ in TSPO PET was significantly associated with OS (*P* = 0.023), with high SUV_max_ of more than 2.2 leading to a hazard ratio of 2.212 (95% CI, 1.115–4.386) for death. The results of the multivariate analyses are presented in Table 4.

**TABLE 4.** Multivariate Survival Analyses

	OS		PFS	
Parameter	Hazard ratio	Significance*	Hazard ratio	Significance*
Age	2.573 (1.284–5.156)	***P* = 0.008**	2.604 (1.341–5.057)	***P* = 0.005**
MGMT promoter methylation status	3.174 (1.368–7.363)	***P* = 0.007**	2.695 (1.266–5.737)	***P* = 0.010**
T2w MRI volume	1.896 (0.993–3.619)	*P* = 0.053	—	—
SUV_max_	2.212 (1.115–4.386)	***P* = 0.023**	—	—

* Bold font highlights the statistically significant associations.

T2w = T2-weighted.

Data in parentheses are 95% CI.

## DISCUSSION

In this study, we corroborate TSPO as a promising imaging target in glioblastoma because tumoral TSPO radioligand uptake in PET appears to be associated with prognosis of glioblastoma patients. In a homogeneous cohort of patients with newly diagnosed IDH–wild-type glioblastoma undergoing TSPO PET before radiotherapy, high tumoral uptake was associated with shorter OS, independent of known clinical risk factors for short survival.

A potential prognostic relevance of TSPO in glioma was proposed more than 25 y ago (11). Multiple efforts have been made to better understand the role of TSPO in glioblastoma at a pathophysiologic level, and now, the potential prognostic relevance of TSPO seems to integrate more coherently into the overall picture of TSPO as a relevant functional player in glioblastoma (2). Recently, we found a TSPO PET signal to be associated with survival in patients with recurrent glioma (12). However, data on the potential prognostic relevance of TSPO PET in newly diagnosed IDH–wild-type glioblastoma were missing until now.

In the current study, patients with high SUV_max_ greater than the median of 2.2 in TSPO PET survived for a significantly shorter period than patients with lower tumoral uptake (OS, 8.3 vs. 17.8 mo; *P* = 0.037). This association persisted when performing multivariate analysis that included prognostic clinical factors: compared with patients with low tumoral uptake on TPSO PET, patients with SUV_max_ of more than 2.2 had a significantly higher risk for death, with a hazard ratio of 2.2 in the multivariate analysis (*P* = 0.023).

Contrast enhancement on MRI was absent for 5 patients, all of whom showed relatively low tumoral uptake in PET and a tendency toward longer OS. Although lack of blood–brain barrier disruption may have been the mechanistic cause for the low uptake in terms of low tracer delivery in those patients, the altered blood–brain barrier passage instead might be merely an epiphenomenon of a diverging biology of the tumor microenvironment with inherently diverging TSPO expression (13*,*^14^). Because the relationship between specific TSPO radioligand uptake and blood–brain barrier disruption has already been the subject of vivid discussion (15), the latter hypothesis may provide an outlet for further research on the underlying pathophysiology, with the potential to better understand the temporal evolution of contrast enhancement and TSPO expression in the context of disease progression. Apart from this association, groups of patients with low and with high tumoral uptake on TSPO PET did not significantly differ with regard to known clinical prognosticators. All 6 LAB cases were among patients with high tumoral uptake in PET. In a previous study, it was reported that LAB status is associated with survival in male glioblastoma patients; in the current study, subgroups were too small to investigate the sex-specific impact of LAB status on survival (16). Yet TSPO binding affinity status, as assessed by genotyping, was not associated with survival in the overall group, either with PFS or with OS.

Apart from SUV_max_ on TSPO PET, parameters associated with survival were age, *MGMT* promoter methylation status, and T2-weighted MRI tumor volume. Patients with higher SUV_max_ did not exhibit significantly larger tumor volumes on MRI, rendering partial-volume effects or SUV_max_ as a confounding surrogate for tumor volume improbable (e.g., there was no correlation of SUV_max_ with T2-weighted MRI volume, *r* = 0.025, *P* = 0.872). As addressed earlier, lack of contrast enhancement on MRI showed a tendency toward longer OS (*P* = 0.070). Historically, contrast enhancement on MRI is an established sign of malignancy in gliomas, which would in principle fit this finding—although the association between contrast enhancement and clinical outcome is continuously critically revisited, embracing resurgence of the topic of the appropriate extent of surgical resection in glioma with regard to non–contrast-enhancing tumor parts (17*,*^18^). The statistical significance of this association in the present study might potentially have been missed because of the inherently small number of non–contrast-enhancing glioblastomas (*n* = 5 vs. 40). The mode of radiotherapy also showed a tendency toward an association with OS, with a better outcome in the group receiving combined normofractionated radiochemotherapy (16.3 vs. 6.8 mo, *P* = 0.105). This can be partly related to receipt of concomitant temozolomide by all patients under the normofractionated radiotherapy regimen but by only 13 of 22 patients in the course of hypofractionated radiotherapy. Still, in a subgroup analysis of the patients undergoing hypofractionated radiotherapy, a trend toward superior survival could be observed depending on the administration of temozolomide, but no significant survival difference was observed as would have been expected—for example, *P* = 0.064 for OS versus *P* < 0.001 in the randomized study of Perry et al. (19). The latter association reveals the limitation of a low case number for distinct subgroups. Instead, in line with the literature, the radiotherapy mode may be associated with clinical outcome because less frail patients were selected for the normofractionated regimen according to clinical routine (9*,*^19^*,*^20^). This is also valid for the patients included in this study, who were significantly younger (59.1 vs. 73.7 y, *P* < 0.001) and functionally less impaired (Karnofsky performance status scale group difference, *P* = 0.003). Within the group of patients with conventional chemoradiotherapy, longer PFS could be observed in those receiving adjuvant temozolomide. This may be attributable partly to undertreatment of patients in whom temozolomide could not be administered (e.g., because of thrombocytopenia) but also may relate to a certain selection bias, because patients with early progression during chemoradiotherapy did not receive adjuvant temozolomide. In the overall group, the percentage of patients with unmethylated *MGMT* promoter (73.3%) was higher than in most MGMT landmark trials and therefore might have contributed to the rather short OS of 10.8 mo in our study cohort (20–22). Patient age and *MGMT* status were significantly associated with both OS and PFS. In contrast, SUV_max_ was significantly associated only with OS, not with PFS. Although we used criteria defined by the Response Assessment in Neuro-Oncology Working Group for progression, this may be partly caused by progression constituting a continuum, rather than a definite point of time (10). The accurate capture of progression depends on imaging time points and is especially difficult in this case because most patients received only a biopsy, not a tumor resection. Conversely, OS is a well-defined, hard endpoint and therefore the more rigid and preferred parameter for survival analyses (10).

Beyond the limitation of the retrospective study design and a rather small sample size regarding distinct subgroups, as elucidated earlier, further points are worth discussing. A benefit of PET imaging for survival prediction in glioma has already been shown using the amino acid analog [^18^F]FET, which is an established PET imaging tracer for gliomas (23). Therefore, inclusion of [^18^F]FET PET data into the current study would have been interesting. Unfortunately, most patients received [^18^F]FET PET later in the disease course, and dual-tracer PET before radiotherapy was not available in a large number of cases in this cohort. Intentionally, this study addressed the association of TSPO PET findings and survival in newly diagnosed glioblastoma before radiotherapy (not during or after radiotherapy, where radiation treatment–related alterations of tumoral tracer uptake may occur in TSPO PET) (24). However, serial TSPO PET imaging during radiotherapy will be of interest as a potential tool for treatment response assessment. In particular, dual-tracer approaches using amino acid and TSPO PET, as well as the monitoring of changes of TSPO radioligand uptake in glioblastoma patients undergoing radiotherapy, harbor a chance of allowing a better understanding of the role of TSPO in the frame of radiotherapy. Recently, we illustrated the potential value of such an approach in a case of an IDH–wild-type glioma with remarkably long survival in the context of chemoradiotherapy. The distinct uptake patterns in dual PET over the disease course in this case led us to speculate that serial TSPO PET, in conjunction with [^18^F]FET PET, might capture the treatment-induced immune response as a potential biomarker (25). In addition, some promising preclinical studies hint at the potential future clinical value of multitracer approaches for PET imaging of glioma. Pigeon et al. revealed TSPO to be an earlier marker for glioma infiltration (26). Foray et al. (27) and Zinnhardt et al. (28) substantiated that each imaging biomarker might identify distinct areas of the heterogeneous glioma tissue and tumor microenvironment; for example, TSPO indicated specific areas of myeloid cell infiltration. Foray et al. used a dual-tracer TSPO and FET approach to image glioma-associated microglia and macrophage dynamics under immunomodulating treatment (29). Further studies are warranted and will enhance the evaluation of TSPO PET for prognostication in glioma patients.

## CONCLUSION

High tumoral uptake in TSPO PET before radiotherapy is associated with significantly shorter survival within the homogeneous group of molecularly defined, newly diagnosed IDH–wild-type glioblastoma. TSPO PET seems to add prognostic insights beyond established clinical parameters and might serve as an informative tool as clinicians make survival predictions for patients with glioblastoma.

## DISCLOSURE

Funding was provided by the Deutsche Forschungsgemeinschaft (DFG), Research Unit FOR 2858, project numbers 421887978 and 422188432. Nathalie Albert was funded by Else Kröner-Fresenius-Stiftung. Matthias Brendel was funded by the DFG under Germany’s Excellence Strategy within the framework of the Munich Cluster for Systems Neurology (EXC 2145 SyNergy 390857198). No other potential conflict of interest relevant to this article was reported.

**KEY POINTS**

**QUESTION:** Is a TSPO PET signal associated with survival in glioblastoma patients?

**PERTINENT FINDINGS:** In a homogeneous cohort of patients with newly diagnosed IDH–wild-type glioblastoma before radiotherapy, a TSPO PET signal was associated with survival. High uptake intensity (SUV_max_ > 2.2) was related to significantly shorter OS. In the multivariate survival analysis, SUV_max_ in TSPO PET remained an independent prognostic factor for OS.

**IMPLICATIONS FOR PATIENT CARE:** TSPO PET seems to add prognostic insights beyond established clinical parameters and might serve as an informative tool as clinicians make survival predictions for patients with glioblastoma.
